# No medication prescription and residential distance from the hospital are important factors associated with nonsurgical weight-loss treatment discontinuance in Japanese patients with high-degree obesity: a retrospective study

**DOI:** 10.1186/s12913-024-11474-2

**Published:** 2024-09-16

**Authors:** Masahiro Ohira, Sayaka Tsuji, Yasuhiro Watanabe, Kazuki Abe, Shuhei Yamaoka, Shoko Nakamura, Rena Oka, Shou Tanaka, Naoyuki Kawagoe, Takashi Yamaguchi, Daiji Nagayama, Ichiro Tatsuno, Atsuhito Saiki

**Affiliations:** 1 Division of Diabetes, Metabolism and Endocrinology, Department of Internal medicine, Toho University Ohashi Medical Center, 2-22-36 Ohashi, Meguro-ku, Tokyo, 153-8515 Japan; 2 Center for Diabetes, Endocrine and Metabolism, Toho University Sakura Medical Center, Sakura-city, Chiba Japan; 3 Nagayama Clinic, Oyama-city, Tochigi Japan; 4 Chiba Prefectural University of Health Sciences, Chiba-city, Chiba Japan

**Keywords:** High-degree obesity, Dropout, Medication, Residential distance, Telemedicine

## Abstract

**Background:**

Although the percentage of the population with a high degree of obesity (body mass index [BMI] ≥ 35 kg/m^2^) is low in Japan, the prevalence of obesity-related diseases in patients with high-degree obesity is greater than that in patients with a BMI < 35 kg/m^2^. Therefore, treatment for high-degree obesity is important. However, clinical studies have reported that 20–50% of patients with obesity discontinue weight-loss treatment in other countries. The circumstances surrounding antiobesity agents are quite different between Japan and other countries. In this study, we investigated the predictors of treatment discontinuation in Japanese patients with high-degree obesity.

**Methods:**

We retrospectively reviewed the medical charts of 271 Japanese patients with high-degree obesity who presented at Toho University Sakura Medical Center for obesity treatment between April 1, 2014, and December 31, 2017. The patients were divided into non-dropout and dropout groups. Patients who discontinued weight-loss treatment within 24 months of the first visit were defined as “dropouts.” Multivariate Cox proportional hazards regression analysis and Kaplan–Meier survival analysis were performed to examine the factors predicting treatment withdrawal.

**Results:**

Among the 271 patients, 119 (43.9%) discontinued treatment within 24 months of the first visit. The decrease in BMI did not significantly differ between the two groups. No prescription of medication and residential distance from the hospital exceeding 15 km were the top contributors to treatment discontinuation, and the absence of prescription medication was the most important factor. The dropout-free rate was significantly higher in patients with medication prescriptions than in those without and in patients who lived within 15 km of the hospital than in those who lived farther than 15 km from the hospital.

**Conclusions:**

No medication prescription and longer residential distance from the hospital were associated with treatment dropout in Japanese patients with high-degree obesity; therefore, the addition of antiobesity medications and telemedicine may be necessary to prevent treatment discontinuation in such patients.

**Supplementary Information:**

The online version contains supplementary material available at 10.1186/s12913-024-11474-2.

## Introduction

Obesity and obesity-related diseases are global health issues. The Japan Society for the Study of Obesity (JASSO) defines obesity as a BMI of 25 kg/m^2^ or greater [1]. In Japan, BMI values are 25 or greater in 24% of the population, 30 or greater in 3%, 35 or greater in 0.5%, and 40 or greater in 0.2% [2], and the rate of high-degree obesity (body mass index [BMI] ≥ 35 kg/m^2^) [1] is lower than that in Europe or the United States [2]. However, the World Health Organization Expert Committee reported that many Asian countries have a low prevalence of obesity but high rates of obesity-related diseases [3]. Indeed, the BMI cutoffs for obesity-related diseases such as diabetes mellitus (DM), hypertension, dyslipidemia, and hyperuricemia are lower in Japanese patients than in American patients [4]. Furthermore, the prevalence of DM, hypertension, and hyperuricemia in Japanese patients with high-degree obesity is greater than that in Japanese patients with a BMI < 35 kg/m^2^ [4]; therefore, treatment for those with high-degree obesity is important.

In Japan, laparoscopic sleeve gastrectomy for high-degree obesity has been covered by National Health Insurance since 2014 and has shown excellent therapeutic effects [5]. However, not all patients with high-degree obesity select or have surgical indication to bariatric surgery. Although the therapeutic effects of nonsurgical weight-loss treatment are inferior to those of bariatric surgery, nonsurgical weight-loss treatment improves weight and obesity-related diseases for at least three years [6]. Considering the necessity for obesity treatment, 20 to 50% of patients with obesity discontinue weight-loss treatment in other countries [7–9]. In Japan, the central issue of obesity treatment may be pharmacotherapy. Mazindol is the only anti-obesity agent approved for high-degree obesity and is covered by the national health insurance in Japan [10–12]. Evidently, if patients with obesity have concomitantly developed type 2 diabetes, glucagon-like peptide-1 (GLP-1) receptor agonist and sodium-glucose co-transporter-2 (SGLT-2) inhibitors, which have weight-loss effects, can be prescribed. In contrast, medications other than mazindol cannot be prescribed even in cases of high-degree obesity not complicated with type 2 diabetes. Discontinuing weight-loss treatment results in missed opportunities to improve body weight and ameliorate obesity-related diseases. In Japan, the issue of pharmacotherapy in patients with high-degree obesity may lead to discontinuation of nonsurgical weight-loss therapy, but no studies predicting these factors with respect to Japanese patients with high-degree obesity have been reported.

Predicting the factors of treatment withdrawal may aid in increasing the treatment continuation rate, and continuing weight-loss treatment may maintain better physical conditions in patients with high-degree obesity. The aim of this study was to investigate the factors predicting treatment dropout in Japanese patients with high-degree obesity who received nonsurgical weight-loss treatment.

## Materials and methods

### Ethics approval and consent to participate

The study protocol was prepared in accordance with the Declaration of Helsinki and approved by the Ethics Committee of Toho University Sakura Medical Center (approval date: August 9, 2021; approval number: S21023). The need for informed consent was waived owing to the retrospective nature of this study by the Ethics Committee of Toho University Sakura Medical Center. Potential participants were allowed to decline participation or withdraw from the study.

### Study design and participants

We retrospectively reviewed the clinical data of patients with high-degree obesity (BMI ≥ 35 kg/m^2^ at the first visit) who presented for obesity treatment to the ambulatory care for obesity clinic at Toho University Sakura Medical Center (Sakura Hospital) between April 1, 2014, and December 31, 2017. Patients who dropped out of weight-loss treatment were identified. “Dropout” was defined as a patient who did not appear for over 4 months from the appointment and discontinued weight-loss treatment within 24 months from the first visit. The study period was determined based on the following factors: SGLT-2 inhibitors were approved and marketed in Japan in April 2014, and the last follow-up date was just before the outbreak of the coronavirus disease (COVID-19) in December 2019. Some clinical studies have reported that body weight decreases in the first 6 or 12 months after obesity treatment but increases again after 12 months [13–15]. We considered that the second year of obesity treatment is important and, therefore, set 24 months as the observation period. Patients with high-degree obesity were selected because prescription of mazindol was possible. We excluded patients who moved to other medical facilities, those who discontinued visits to Sakura Hospital for known reasons, those with secondary obesity, those whose body weight was not recorded in the medical chart, and patients who died within 24 months of the first visit. Additionally, we excluded patients who underwent bariatric surgery within 24 months of the first visit because bariatric surgery is a more effective weight-loss therapy than nonsurgical treatments are [5, 6], which may motivate patients to continue hospital visits, and the target of this study was patients with high-degree obesity who received nonsurgical weight-loss treatment.

### Weight-loss treatment

Patients were followed monthly and instructed by a physician regarding lifestyle modifications, which included reducing energy intake, increasing physical activity, measuring body weight daily, and recording this data in a specified weight control notebook to be checked by the physician at follow-up. Patients were also instructed by a physician or dietitian to follow a diet therapy in accordance with 20–25 kcal/kg of ideal body weight per day. Physicians usually explain and propose bariatric surgery to patients with high-degree obesity at the first visit and at least every 6 months. When patients do not select bariatric surgery, physicians continue nonsurgical treatment. A prescription of mazindol is considered when the patient’s body weight does not decrease by 3% for 3 months, but the prescription of mazindol is limited to a maximum of 2 weeks per prescription, with a maximum treatment period of 3 months in Japan. GLP-1 receptor agonists and/or SGLT-2 inhibitors are prescribed for body weight reduction, as mentioned above, and blood glucose control in patients with high-degree obesity complicated with type 2 diabetes. GLP-1 receptor agonists can be prescribed only in patients with type 2 diabetes because of the rules of Japanese National Health Insurance.

### Definitions of clinical factors

The definitions of body weight and BMI records, type 2 diabetes, lipid disorders, hyperuricemia, hypertension, mental health disease, prescriptions, continuous nutritional guidance, hospitalization, visit to other department, and residential distance were as follows.

Body weight and BMI at last visit were measured at the last visit of dropout patients and at the follow-up visit after 24 months of treatment in non-dropout patients. Body weight and BMI changes between the first and last visits were calculated as the difference in measurements between the first visit and the last visit in dropout patients or between the first visit and the follow-up visit at 24 months of treatment in non-dropout patients. The lowest body weight and BMI were defined as the lowest body weight and BMI, respectively, achieved during the observation period post weight loss treatment; these changes were not observed in the patients who discontinued treatment after the first visit. Thirty-three patients (27.7% in the dropout group) dropped out after the first visit. These patients had no data after the second visit. Data on weight and BMI (at the second visit, at last visit, lowest, and changes between the first visit and those) were required at least two visits to calculate these results; 33 patients dropped out after the first visit, the numbers of these data decreased from 271 to 238 (minus 33 patients) for all patients (Tables 1 and 2) and from 119 to 86 (minus 33 patients) in the dropout group (Table 3).

**Table 1:** Patient characteristics

No. of patients analyzed No. of dropouts Age at the first visit (years) Sex (male/female) Height (cm)	271 119 (43.9%) 41.0 (32.0–50.0) 150 (55.4%)/121 (44.6%) 167.0 (159.0–174.0)
Body weight (kg)	
at first visit at second visit (*n* = 238) at last visit (*n* = 238) lowest (*n* = 238) change between first and last visit (*n* = 238) change between first and second visit (*n* = 238) change between first visit and lowest (*n* = 238)	114.0 (100.0–130.6) 111.2 (96.4–127.4) 108.6 (94.5–125.8) 105.4 (90.8–120.9) -5.3 (-10.7–-0.5) -2.7 (-5.3–-0.8) -7.9 (-13.0–-3.5)
BMI (kg/m^2^)	
at first visit at second visit (*n* = 238) at last visit (*n* = 238) lowest (*n* = 238) change between first and last visit (*n* = 238) change between first and second visit (*n* = 238) change between first visit and lowest (*n* = 238)	40.8 (36.5–46.6) 39.6 (35.5–45.0) 38.9 (34.4–44.3) 37.8 (33.4–42.9) -2.0 (-4.0–-0.2) -1.0 (-1.9–-0.3) -2.9 (-5.1–-1.2)
Complications	
Type 2 diabetes (%) Hypertension (%) Lipid disorders (%) Hyperuricemia (%) Thyroid disease (%) Mental health disease (%)	141 (52.0%) 127 (46.9%) 139 (51.3%) 59 (21.8%) 12 (4.4%) 77 (28.4%)
Prescription from the Diabetes Center (%) Prescription of mazindol (%) Prescription of medications with weight-loss effect (%) Prescription of medications for diseases in the specialties (%) Prescription of medications for other chronic diseases (%)	137 (50.6%) 15 (5.5%) 69 (25.5%) 124 (45.8%) 55 (20.3%)
Continuous nutritional guidance (%) Hospitalization (%) Visit to other departments in Sakura Hospital (%)	203 (74.9%) 51 (18.8%) 74 (27.3%)
Distance from residence to Sakura Hospital	
≤15 km >15 km	144 (53.1%) 127 (46.9%)

Data are presented as median and interquartile range (IQR) or numbers

BMI, body mass index; lowest, lowest body weight and BMI noted during observation period

Diabetes Center = The Center for Diabetes, Endocrine, and Metabolism, Toho University Sakura Medical Center. Prescription of medications for diseases of the specialties = prescription of medications for obesity, diabetes, hypertension, hyperlipidemia, hyperuricemia, and/or endocrine disease. Sakura Hospital = Toho University Sakura Medical Center

**Table 2:** Correlation between dropout (no, 0; yes, 1) and clinical factors

	Dropout (0, no; 1, yes)	
	ρ	*P* value
Age at first visit (years) Sex (0, male; 1, female) Height (cm)	-0.0892 0.0878 -0.0506	0.1431 0.1497 0.4065
Body weight (kg)		
at first visit at second visit (*n* = 238) at last visit (*n* = 238) lowest (*n* = 238) change between first and last visit (*n* = 238) change between first and second visit (*n* = 238) change between first visit and lowest (*n* = 238)	-0.0815 -0.1175 -0.0232 -0.0457 0.0609 -0.0678 0.1484	0.1812 0.0704 0.7039 0.4828 0.3519 0.2976 0.0220
BMI (kg/m^2^)		
at first visit at second visit (*n* = 238) at last visit (*n* = 238) lowest (*n* = 238) change between first and last visit (*n* = 238) change between first and second visit (*n* = 238) change between first visit and lowest (*n* = 238)	-0.0735 -0.1029 0.0016 -0.0128 0.0585 -0.0735 0.1451	0.2280 0.1135 0.9794 0.8443 0.3707 0.2590 0.0252
Complications		
Type 2 diabetes (no, 0; yes, 1) Hypertension (no, 0; yes, 1) Lipid disorders (no, 0; yes, 1) Hyperuricemia (no, 0; yes, 1) Thyroid disease (no, 0; yes, 1) Mental health disease (no, 0; yes, 1)	-0.1178 -0.0114 -0.1344 -0.1965 -0.1543 -0.0958	0.0527 0.8513 0.0269 0.0011 0.0110 0.1156
Prescription from the Diabetes Center (no, 0; yes, 1) Prescription of mazindol (no, 0; yes, 1) Prescription of medication with weight-loss effect (no, 0; yes, 1) Prescription of medication for diseases of the specialties (no, 0; yes, 1) Prescription of medications for other chronic diseases (no, 0; yes, 1)	-0.3890 -0.1817 -0.3806 -0.3500 -0.2986	< 0.0001 0.0027 < 0.0001 < 0.0001 < 0.0001
Continuous nutritional guidance (no, 0; yes, 1) Hospitalization (no, 0; yes, 1) Visit to other departments in Sakura Hospital (no, 0; yes, 1) Residential distance (≤ 15 km, 0; >15 km, 1)	-0.0710 -0.0836 -0.1251 0.1376	0.2440 0.1700 0.0396 0.0235

BMI, body mass index; lowest, lowest body weight and BMI noted during observation period

Diabetes Center = The Center for Diabetes, Endocrine, and Metabolism, Toho University Sakura Medical Center. Prescription of medications for diseases of the specialties = prescription of medications for obesity, diabetes, hypertension, hyperlipidemia, hyperuricemia, and/or endocrine disease. Sakura Hospital = Toho University Sakura Medical Center

**Table 3:** Comparison of patient characteristics between the non-dropout and dropout group

	Non-dropout	Dropout	*P* value
No. of patients	152 (56.1%)	119 (43.9%)	
Age at first visit (years) Sex (male/female) Height (cm)	41.0 (34.3–50.0) 90 (59.2%)/62 (40.8%) 167.8 (159.3–174.0)	39.0 (29.0–49.0) 60 (50.4%)/59 (49.6%) 165.0 (159.0–173.0)	0.1430^a^ 0.1758^b^ 0.4059^a^
Body weight (kg)			
at first visit at second visit at last visit lowest change between first and last visit change between first and second visit change between first visit and lowest	116.0 (100.0–133.3) 113.5 (98.7–-131.2) 109.1 (94.6–128.3) 105.9 (91.6–124.7) -6.5 (-11.1–-0.6) -2.6 (-5.1–-0.6) -8.4 (-14.5–-4.2)	110.0 (100.0–129.4) 106.8 (93.1–121.9) 108.1 (93.9–123.4) 103.3 (86.8–118.5) -4.7 (-9.4–-0.5) -3.2 (-6.7–-0.9) -6.0 (-11.5–-2.5)	0.1810^a^ 0.0706^a^ 0.7073^a^ 0.4823^a^ 0.3513^a^ 0.2971^a^ 0.0224^a^
BMI (kg/m^2^)			
at first visit at second visit at the last visit lowest change between first and last visit change between first and second visit change between first visit and lowest	41.2 (36.9–46.7) 39.9 (35.9–45.3) 38.9 (34.4–44.2) 38.0 (33.4–43.2) -2.4 (-4.1–-0.2) -0.9 (-1.7–-0.2) -3.1 (-5.3–-1.5)	40.2 (35.9–45.8) 38.5 (34.1–44.5) 38.9 (34.4–44.4) 37.7 (33.1–42.8) -1.6 (-3.5–-0.2) -1.2 (-2.3–-0.3) -2.2 (-4.5–-0.9)	0.2276^a^ 0.1135^a^ 0.9799^a^ 0.8446^a^ 0.3701^a^ 0.2585^a^ 0.0255^a^
Complications			
Type 2 diabetes (%) Hypertension (%) Lipid disorders (%) Hyperuricemia (%) Thyroid disease (%) Mental health disease (%)	87 (57.2%) 72 (47.4%) 87 (57.2%) 44 (28.9%) 11 (7.2%) 49 (32.2%)	54 (45.4%) 55 (46.2%) 52 (43.7%) 15 (12.7%) 1 (0.8%) 28 (23.5%)	0.0660^b^ 0.9026^b^ 0.0283^b^ 0.0017^b^ 0.0143^b^ 0.1358^b^
Prescription from the Diabetes Center (%) Prescription of mazindol (%) Prescription of medications with weight-loss effect (%) Prescription of mediations for diseases of the specialties (%) Prescription of medications for other chronic diseases (%)	103 (67.8%) 14 (9.2%) 61 (40.1%) 93 (61.2%) 47 (31.0)	34 (28.6%) 1 (0.8%) 8 (6.7%) 31 (26.1%) 8 (6.7)	< 0.0001^b^ 0.0023^b^ < 0.0001^b^ < 0.0001^b^ < 0.0001^b^
Continuous nutritional guidance (%) Hospitalization (%) Visit to other department in Sakura Hospital (%)	118 (77.6%) 33 (21.7%) 49 (32.2%)	85 (71.4%) 18 (15.1%) 25 (21.0%)	0.2609^b^ 0.2104^b^ 0.0408^b^
Distance from residence to Sakura Hospital			0.0274^b^
≤15 km >15 km	90 (59.2%) 62 (40.8%)	54 (45.4%) 65 (54.6%)	

^a^Wilcoxon rank-sum test, ^b^Fisher’s exact test. Data are presented as median and interquartile range (IQR) or numbers. The number of patients with changes in body weight and BMI at second and last visit and who achieved lowest body weight and BMI (lowest) during the observation period were 86 (dropout group)

BMI, body mass index. Diabetes Center = the Center for Diabetes, Endocrine, and Metabolism, Toho University Sakura Medical Center. Prescription of medications for diseases of the specialties = prescription of medications for obesity, diabetes, hypertension, hyperlipidemia, hyperuricemia, and/or endocrine disease

Sakura Hospital = Toho University Sakura Medical Center

Other conditions such as type 2 diabetes, lipid disorders, and hyperuricemia were identified in patients by assessing their prescriptions of current medication and laboratory data. Type 2 diabetes was confirmed at fasting blood glucose concentration ≥ 126 mg/dL, casual blood glucose ≥ 200 mg/dL, and glycated hemoglobin (HbA1c) ≥ 6.5%; lipid disorder at low-density lipoprotein cholesterol ≥ 140 mg/dl, fasting triglyceride ≥ 150 mg/dl, non-fasting triglyceride ≥ 175 mg/dl [16], and/or high-density lipoprotein cholesterol < 40 mg/dl; hyperuricemia at uric acid > 7.0 mg/dl; and hypertension at systolic blood pressure (BP) > 140 mmHg and/or diastolic BP > 90 mmHg and when patients were receiving antihypertensive drugs. Patients were considered to have developed these diseases only if patients met these criteria at least once during the observation period. Patients who were already cared for by psychiatrists at the first visit or who complained of mental health symptoms and consulted with psychiatrists after they started weight-loss treatment were considered to have mental health diseases.

All “prescriptions” refer to the prescriptions of medications issued by the Center for Diabetes, Endocrine and Metabolism (Diabetes Center) during the observation period. “Prescription from Diabetes Center” refers to the prescription of at least one medication for chronic disease. “Prescription of medication with weight-loss effect” refers to the prescription of GLP-1 receptor agonist, SGLT-2 inhibiter, and/or mazindol. “Prescription of medications for diseases of the specialties” refers to the prescription of medications for obesity, diabetes, hypertension, hyperlipidemia, hyperuricemia, and/or endocrine disease. “Prescription of medications for other chronic diseases” refers to the prescription of medications for diseases other than the specialties, such as digestive disease, respiratory disease, allergic diseases, and insomnia.

“Continuous nutritional guidance” refers to the nutritional guidance provided by a dietitian at least once every two visits during the observation period. “Hospitalization” refers to admission to Sakura Hospital for treatment of obesity or/and diabetes during the observation period. “Hospitalizations for treatment of other diseases” were not included in this category. “Visit to other department” refers to the regular visits to other departments in Sakura Hospital. We categorized patients into two groups based on the distance between their residence and Sakura Hospital: within 15 km and over 15 km. Because neighboring cities are located within a 15 km radius from Sakura Hospital, patients living in neighboring cities can easily access the hospital.

### Statistical analysis

Normality of data distribution was tested using the Shapiro–Wilk test. Continuous data are expressed as median and interquartile range (IQR) because some data were not normally distributed. Data from independent samples were analyzed using the Wilcoxon rank-sum test. Fisher’s exact test was used to compare the proportions and categorical variables. The correlations between dropout (no, 0; yes, 1) and each clinical factor were assessed with Spearman’s rank correlation coefficients. Multivariate Cox proportional hazards regression analysis was used to identify the associations between dropout (no, 0; yes, 1) and each clinical factor. Clinical factors that correlated significantly with dropout according to the Spearman’s rank correlation coefficient were selected. There was no multicollinearity in any of the clinical factors (Supplementary Table 1). Changes in body weight and BMI between the first and the lowest were omitted from the model because the number of patients differed between these and other factors. Finally, the following variables were entered into the model as covariates: age, sex, lipid disorders, hyperuricemia, thyroid disease, prescription from the Diabetes Center, prescription of mazindol, prescription of medication with weight-loss effect, prescription of medication for specialties diseases, prescription of medications for other chronic diseases, visit to other departments in Sakura Hospital, and residential distance from Sakura Hospital. The results are expressed as hazard ratios with 95% confidence intervals. Kaplan–Meier survival analysis was used to compare the time-to-dropout rate between the two groups and to verify the difference in dropout status between patients with and without prescription from the Diabetes Center and between patients with residential distances within or over 15 km from Sakura Hospital. A two-sided *P* value less than 0.05 was considered significant. All statistical analyses were performed using the JMP software version 14.3 (SAS Institute, Cary, NC, USA).

## Results

**Fig. 1:**
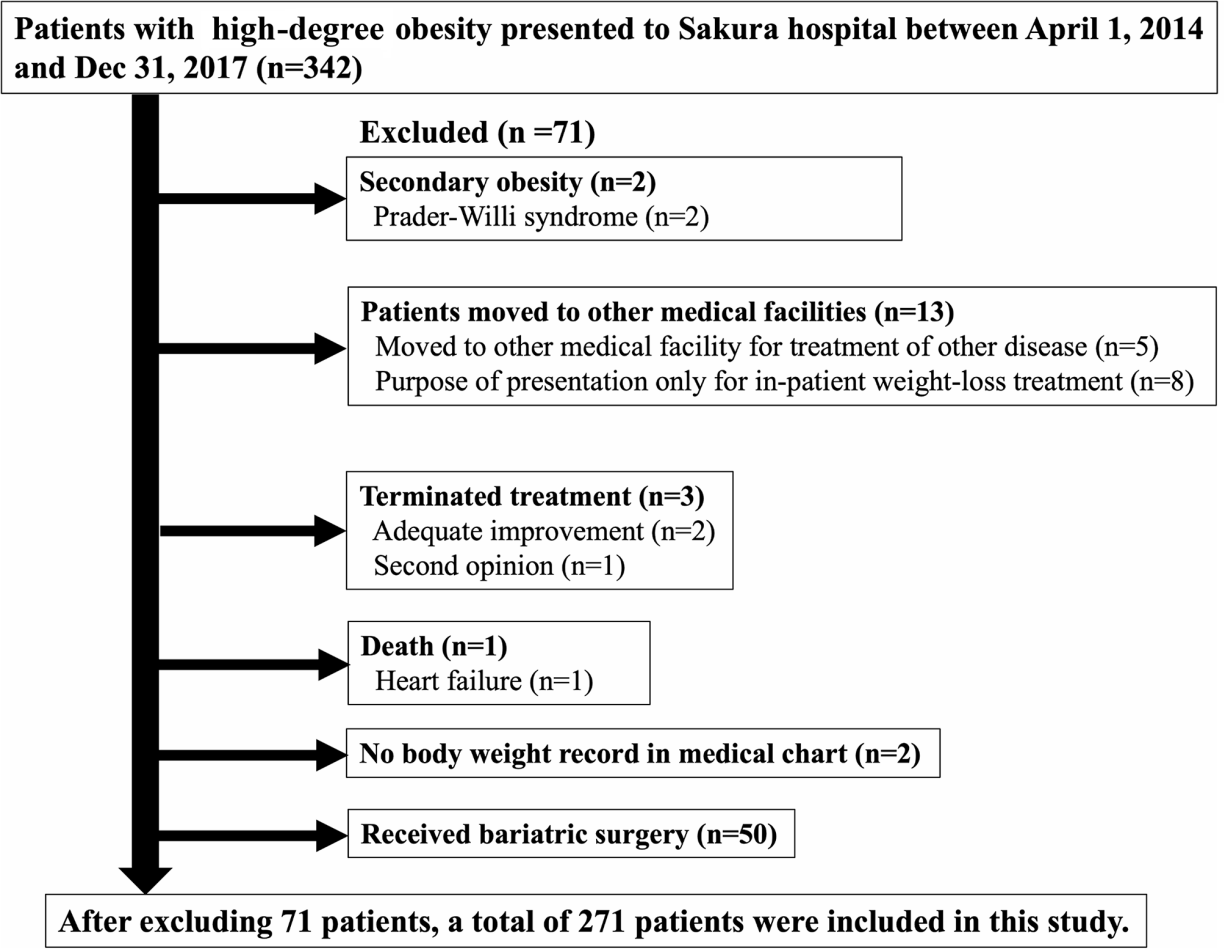
Selection of patients with high-degree obesity A total of 342 patients presented to Toho University Sakura Medical Center for treatment of high-degree obesity between April 1, 2014 and December 31, 2017, out of which, 71 patients were excluded: 2 patients with secondary obesity caused by Prader-Willi syndrome, 13 patients moved to other medical facilities—for treatment of other disease (*n* = 5) and presented to our hospital only for the purpose of hospitalization for weight-loss treatment (*n* = 8), 3 patients terminated treatment owing to adequate improvement (*n* = 2) and second opinion (*n* = 1), 1 patient died within 24 months from the first visit because of heart failure, 2 patients had no body weight record in the medical chart, and 50 patients received bariatric surgery within 24 months from the first visit. A total of 271 patients were included in this study. Adequate improvement means that BMI was decreased to under 25 kg/m^2^ and was maintained within 24 months after starting weight-loss treatment, and two patients terminated treatment upon agreement between a physician and the patient

A total of 342 patients presented to the Toho University Sakura Medical Center for the treatment of high-degree obesity between April 1, 2014, and December 31, 2017. A total of 71 patients were excluded (Fig. 1).

Ultimately, 271 patients (79.2%) were included in this study (Fig. 1). Table 1 shows the characteristics of all the patients in this study. One hundred and nineteen patients (43.9%) dropped out of treatment within 24 months. Although we excluded patients who underwent bariatric surgery within 24 months of the first visit, the dropout rate in these patients was significantly lower than that in patients without bariatric surgery (Supplementary Table 2).

We divided all patients into two groups: non-dropout (*n* = 152, 56.1%) and dropout (*n* = 119, 43.9%) (Table 3). The decrease in body weight and BMI from the first visit to the second or last visit did not differ between the two groups. The duration between the first and second visits did not differ significantly between the 2 groups (Supplementary Table 3), and the median months between the first and last visits was 3 (0–9.0) in the dropout group. In contrast, the decrease in body weight and BMI between the first visit and the lowest was significantly greater in the non-dropout group than in the dropout group (Table 3). The visit at which the lowest body weight and BMI were recorded significantly differed (Supplementary Table 3). The frequencies of lipid disorders, hyperuricemia, and thyroid disease were significantly higher in the non-dropout group than in the dropout group (Table 3). The frequency of prescription from the Diabetes Center, prescription of mazindol, prescription of medications with weight-loss effects, prescription of medications for diseases of the specialties, prescription of medications for other chronic diseases, and visits to other departments in Sakura Hospital were also significantly higher in the non-dropout group than in the dropout group. Compared with patients in the dropout group, larger number of patients in the non-dropout group lived within 15 km of Sakura Hospital. The other factors were not significantly different between the two groups (Table 3).

Lipid disorder, hyperuricemia, thyroid disease, and visits to other departments were negatively correlated with dropout (no, 0; yes, 1). Factors such as body weight and BMI changes between the first visit and the lowest and living beyond 15 km from Sakura Hospital positively correlated with dropout. Prescriptions from the Diabetes Center, prescription of mazindol, prescription of medications with weight-loss effect, prescription of medications for diseases of the specialties, and prescription of medications for other chronic diseases negatively correlated with dropout. Prescriptions from the Diabetes Center had the strongest correlation with dropout (Table 2).

Prescriptions from the Diabetes Center were the strongest contributors to dropout (Table 4). Living over 15 km from Sakura Hospital also contributed to dropout (Table 4).

**Table 4:** Multivariate Cox proportional hazards regression analysis for the association between dropout (no, 0; yes, 1) and clinical factors

	Adjusted Hazard Ratio	95% CI	*P* value
Age at first visit (years) Sex (male, 0; female, 1) Lipid disorders (no, 0; yes, 1) Hyperuricemia (no, 0; yes, 1) Thyroid disease (no, 0; yes, 1) Prescription from the Diabetes Center (no, 0; yes, 1) Prescription of mazindol (no, 0; yes, 1) Prescription of medication with weight-loss effect (no, 0; yes, 1) Prescription of medication for disease of the specialties (no, 0; yes, 1) Prescription of medications for other chronic diseases (no, 0; yes, 1) Visit to other departments in Sakura Hospital (no, 0; yes, 1) Distance from residence to Sakura Hospital (≤ 15 km; 0, > 15 km; 1)	1.001 1.061 0.846 0.721 0.154 0.234 0.527 0.713 0.907 0.986 0.846 2.869	0.986–1.015 0.721–1.563 0.579–1.237 0.495–1.049 0.021–1.117 0.099–0.552 0.064–4.375 0.486–1.044 0.249–3.304 0.266–3.661 0.534–1.339 1.223–6.729	0.9211 0.7621 0.3890 0.0874 0.0642 0.0009 0.5533 0.0821 0.8820 0.9834 0.4752 0.0154

CI: Confidence interval

Diabetes Center = The Center for Diabetes, Endocrine, and Metabolism, Toho University Sakura, Medical Center. Sakura Hospital = Toho University Sakura Medical Center

**Fig. 2:**
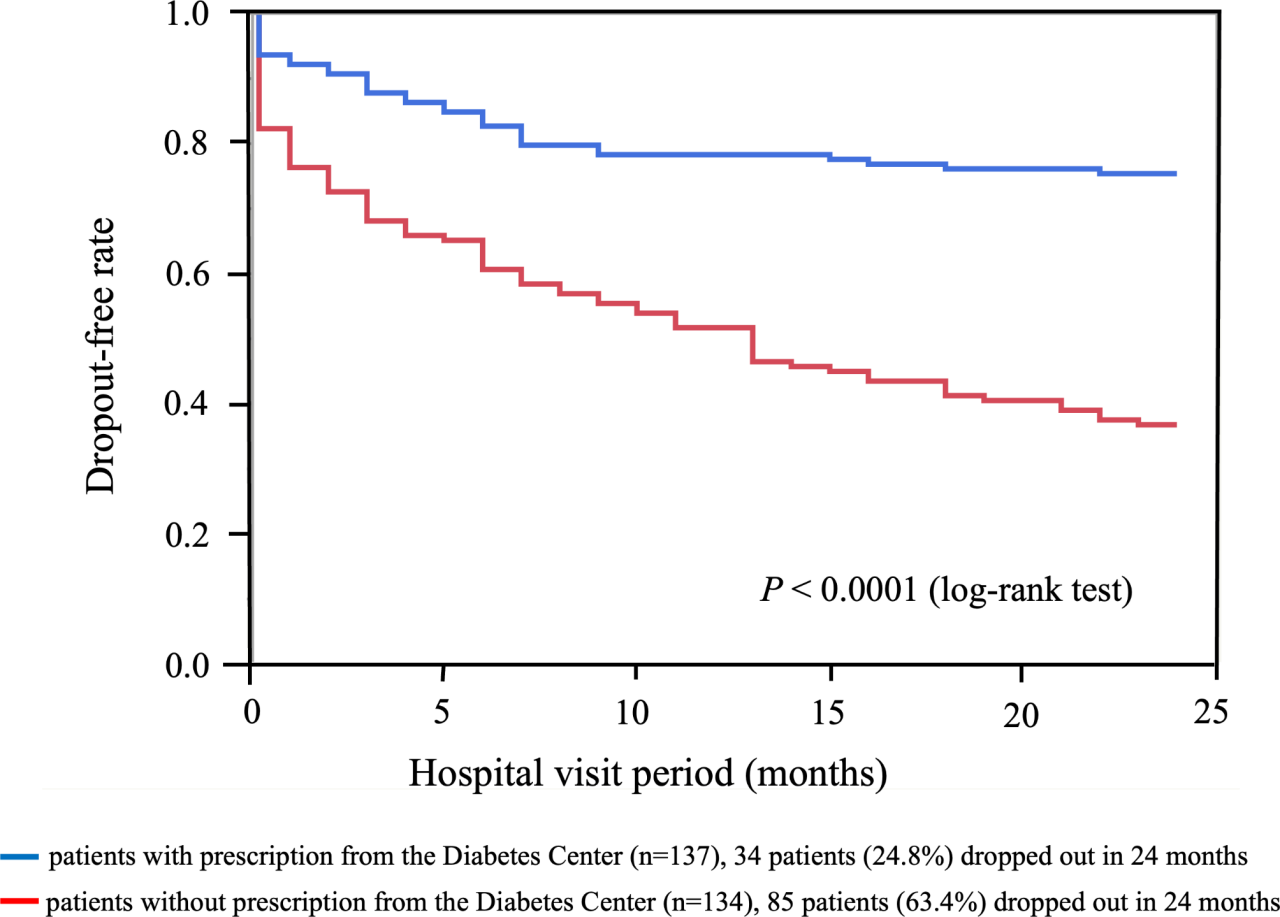
Dropout-free rate by Kaplan–Meier method for patients with/without prescription from Diabetes Center “Dropout” was defined as discontinuation of weight-loss therapy within 24 months from the first visit. Blue and red lines denote patients with (*n* = 137) and without (*n* = 134) prescription from the Diabetes Center, respectively. Cumulatively, 34 patients with and 85 patients without prescription dropped out in 24 months. Dropout event-free rate was significantly higher (*P* < 0.0001) in patients with prescription from the Diabetes Center than in those without. Diabetes Center = the Center for Diabetes, Endocrine and Metabolism, Toho University Sakura Medical Center

**Fig. 3:**
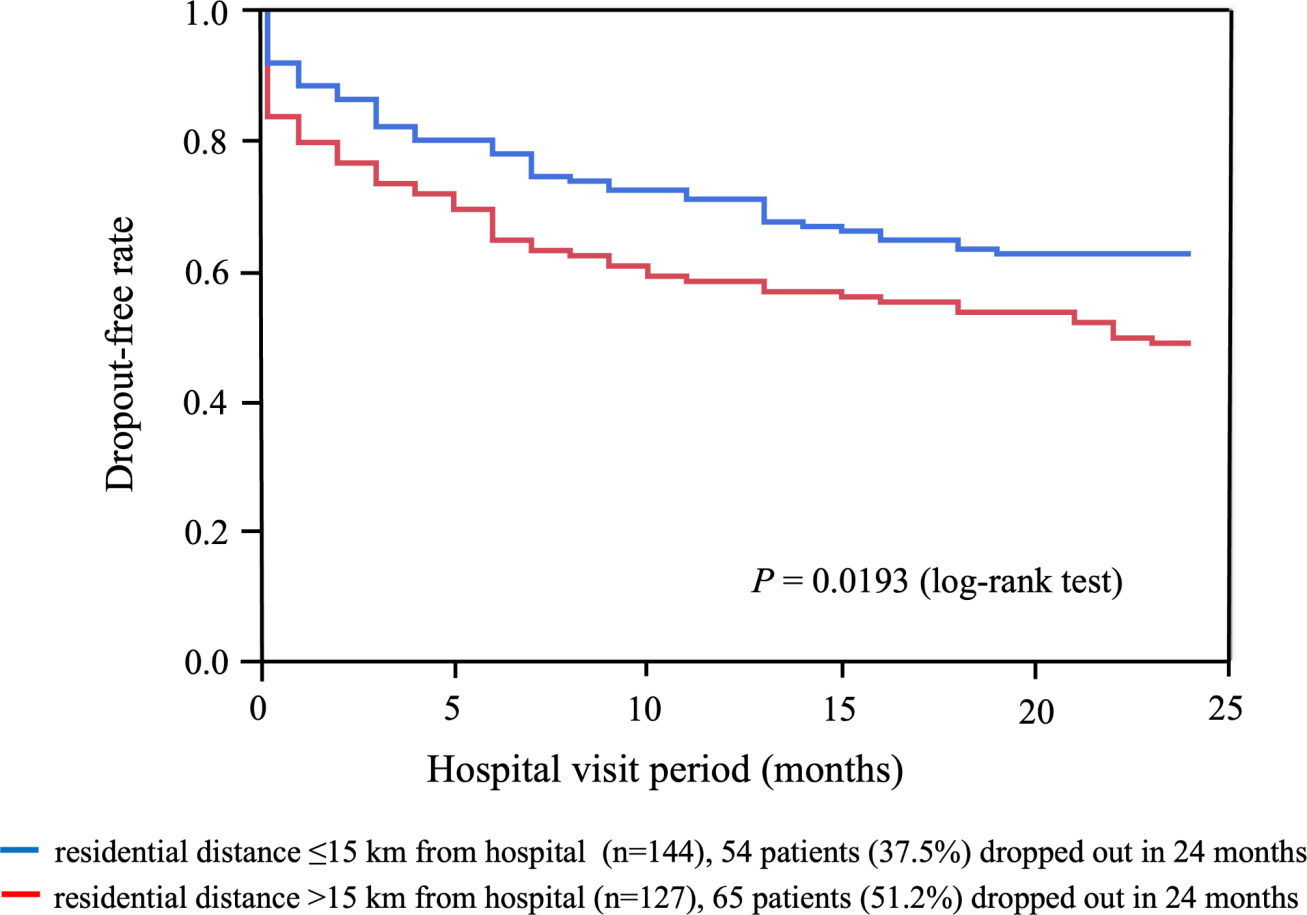
Dropout-free rate by Kaplan–Meier method for patients living within/over 15 km from Sakura Hospital “Dropout” was defined as discontinuation of weight-loss therapy within 24 months from the first visit. Blue and red lines represent patients who lived within (*n* = 144) and over (*n* = 127) 15 km from Sakura Hospital, respectively. Cumulatively, 54 patients who lived within and 65 patients who lived over 15 km from Sakura Hospital dropped out in 24 months. Dropout event-free rate was significantly higher (*P* = 0.0193) in patients who lived closer to Sakura Hospital than in those who lived farther. Sakura Hospital = Toho University Sakura Medical Center

The dropout event-free rate was significantly higher in patients with prescriptions from the Diabetes Center than in patients without prescriptions (Fig. 2). The dropout event-free rate was significantly higher in patients who lived within 15 km of Sakura Hospital than in those who lived farther than 15 km from Sakura Hospital (Fig. 3).

## Discussion

The reduction in body weight and BMI between the first visit and the lowest was significantly greater in the non-dropout group than in the dropout group. The frequencies of lipid disorders, hyperuricemia, thyroid disease, prescription of medications, and visits to other departments were significantly higher in the non-dropout group than in the dropout group. Compared with the dropout group, larger number of patients in the non-dropout group lived within 15 km of Sakura Hospital. Decrease in body weight and BMI between the first visit and the lowest, lipid disorder, hyperuricemia, thyroid disease, prescription of medications, visits to other departments, and living within 15 km of Sakura Hospital correlated significantly with no dropout. No prescription of medication and living over 15 km from Sakura Hospital were significantly associated with dropout, and the absence of medical prescription was the most important factor contributing to dropout among Japanese patients with high-degree obesity.

Mazindol effectively reduces body weight by 2.12 to 13.5 kg in patients with obesity [17–19], but there are limitations to prescribing mazindol, as already mentioned in Japan. This situation probably accounts for the very low rate of mazindol use observed in this study. A poor initial treatment response is an independent predictor of dropout from obesity treatment [7, 20]. Although the initial response to treatment did not differ between the two groups, the changes in body weight and BMI between the first visit and the lowest were significantly greater in the non-dropout group. In this study, the proportion of patients who received pharmacological therapy was much lower, probably due to the prescription rules for mazindol and GLP-1 receptor agonists in the Japanese National Health Insurance program. This is one of the serious issues of nonsurgical treatment for patients with high-degree obesity in Japan. If more anti-obesity agents are permitted in Japan, larger number of patients could be treated appropriately with effective medications that would improve the initial treatment response and/or maximum weight reduction and consequently reduce the dropout rate.

This study showed that residential distance from Sakura Hospital was an important factor associated with dropout in Japanese patients with high-degree obesity. A clinical study showed that residential distance from the clinic is independently associated with dropout in patients with high-degree obesity, type 2 diabetes, or fatty liver disease [21]. Larger residential distance from the clinic was associated with non-completion of intensive specialist obesity management, and the mean residential distance from the clinic was 22 km in non-completers and 17 km in completers [21].

One hundred and nineteen patients (43.9%) discontinued weight loss treatment within 24 months. The dropout rates were 12.2%, 25.1%, and 35.1% after the first visit, 6 months, and 12 months, respectively. Other clinical studies showed that dropout rates for weight loss therapy were 20% after the first visit, 21% after 1 month, 30.5% after 10 weeks, 31.0% after 16 weeks, 51.9 to 57% after 6 months, and 32.3 to 77.3% after 12 months [7, 20–28]. The dropout rates in this study were low compared to those reported in other studies. The reasons for treatment withdrawal among patients with obesity include unsatisfactory outcomes, living farther from the clinic or hospital, family and work problems, lack of motivation, poor weight loss, poor response to treatment, lower educational level, higher levels of obesity, health problems other than obesity, younger age, full-time employment, less need for complex care, and fewer obesity-related complications [7, 20–28]. The residential distance to the hospital was consistent with that reported in other studies.

In the present study, no medication prescription was the most important factor associated with the discontinuation of weight loss treatment in Japanese patients with high-degree obesity. Furthermore, changes in body weight and BMI between the first visit and the lowest were significantly greater in the non-dropout group; hence, the degree of weight reduction may also be important for treatment discontinuation. Greater residential distance was also associated with dropout in Japanese patients with high-degree obesity. Telemedicine approaches can reduce body weight in patients with obesity [29, 30], has been covered by the national health insurance in Japan since 2018, and has become increasingly common since the outbreak of COVID-19. The combination of anti-obesity drugs and telemedicine could prevent or, at the least, reduce the rate of treatment discontinuation in Japanese patients with high-degree obesity.

In this study, the frequencies of lipid disorders, hyperuricemia, and thyroid disease were significantly greater in the non-dropout group than in the dropout group. Fewer obesity-related complications were a reason for treatment withdrawal in patients with obesity in a previous study [22], and prescription from the Diabetes Center was related to dropout in this study. The numbers of comorbidities and patients with prescriptions from the Diabetes Center were significantly greater in the non-dropout group than in the dropout group among patients with lipid disorders (Supplementary Tables 4 and 5). The number of patients with prescriptions from the Diabetes Center was significantly greater in the non-dropout group than in the dropout group among patients with hyperuricemia (Supplementary Tables 4 and 5). These factors may influence the difference in the frequencies of lipid disorders and hyperuricemia between the non-dropout and dropout groups. On the other hand, no significant differences were observed in thyroid disease (Supplementary Tables 4 and 5). In this study, we were not able to clarify why the frequencies of lipid disorders, hyperuricemia, and thyroid disease were significantly greater in the non-dropout group than in the control group, but these comorbidities were not included in the multivariate Cox proportional hazards regression analysis. As a result, we considered that the complications of these comorbidities were not related to withdrawal of nonsurgical weight-loss treatment in patients with high-degree obesity.

This study has some limitations. First, a clinical study showed that full-time employment is the best predictor of premature withdrawal from overweight and obesity treatments [22]; however, we did not analyze the patients’ employment status in this study. Second, some clinical studies have reported that family and work problems, lower educational levels, and lack of motivation are associated with dropout in patients with obesity [7, 24–26]. We did not include these factors in our study and, therefore, were unable to compare these factors between the non-dropout and dropout groups. Finally, because this was a single-center retrospective study, the sample size was limited, and the findings may not be generalizable to other areas. Future multicenter studies enrolling a larger number of Japanese patients with obesity from multiple areas are needed. Furthermore, we could not follow whether the patients continued weight-loss treatment in other clinics near patients’ residential areas after they dropped out of Sakura Hospital. In the future, a study using National Health Insurance claims data is needed to determine whether patients entirely discontinue weight-loss treatment. Despite these limitations, we demonstrated that absence of prescription medications (strongest factor for treatment withdrawal) and farther residential distance from the hospital were associated with discontinuation of weight loss treatment in Japanese patients with high-degree obesity.

## Conclusions

No medication prescription and longer residential distance were associated with discontinuation of weight-loss treatment in Japanese patients with high-degree obesity. Physicians must be aware of the treatment withdrawal risk in patients with high-degree obesity who have not been prescribed medications and live further away from the hospital. The addition of anti-obesity agents and telemedicine may prevent treatment dropout in Japanese patients with high-degree obesity.

## Electronic supplementary material

Below is the link to the electronic supplementary material.

Supplementary Material 1

Supplementary Material 2

Supplementary Material 3

Supplementary Material 4

Supplementary Material 5

## Data Availability

The datasets generated and/or analyzed during the current study are not publicly available due to including patients’ IDs in Toho University Sakura Hospital but are available from the corresponding author on reasonable request.

## Abbreviations

BMI: Body mass index
GLP-1: Glucagon-like peptide-1
SGLT-2: Sodium-glucose co-transporter-2
COVID-19: Coronavirus disease
HbA1c: Glycated hemoglobin
BP: Blood pressure
IQR: Interquartile range
