# Supplementary material for: No medication prescription and residential distance from the hospital are important factors associated with nonsurgical weight-loss treatment discontinuance in Japanese patients with high-degree obesity: a retrospective study
Source: BMC Health Serv Res. 2024 Sep 16;24:1078. doi: 10.1186/s12913-024-11474-2 (PMC11407008; doi:10.1186/s12913-024-11474-2)
Supplement: Supplementary file 1 — Supplementary Material 1 [file 12913_2024_11474_MOESM1_ESM.docx]

Supplementary Table 1. Variance inflation factors showing multicollinearity between potential predictors of dropout

| Variable | VIF | 1/VIF |
| --- | --- | --- |
| Lipid disorders  Hyperuricemia  Thyroid disease  Prescription from the Diabetes Center  Prescription of mazindol  Prescription of medication with weight-loss effect  Prescription of medication for disease specialties  Prescription of medication for other chronic diseases  Visit to other department in Sakura Hospital  Residential distance | 1.08  1.05  1.06  7.70  1.36  1.79  6.63  1.45  1.07  1.03 | 0.93  0.95  0.94  0.13  0.74  0.56  0.15  0.69  0.93  0.97 |

VIF, variance inflation factor. Diabetes Center = Center for Diabetes, Endocrine, and Metabolism, Toho University Sakura Medical Center. Sakura Hospital = Toho University Sakura Medical Center.
