# Supplementary material for: No medication prescription and residential distance from the hospital are important factors associated with nonsurgical weight-loss treatment discontinuance in Japanese patients with high-degree obesity: a retrospective study
Source: BMC Health Serv Res. 2024 Sep 16;24:1078. doi: 10.1186/s12913-024-11474-2 (PMC11407008; doi:10.1186/s12913-024-11474-2)
Supplement: Supplementary file 2 — Supplementary Material 2 [file 12913_2024_11474_MOESM2_ESM.docx]

Supplementary Table 2. Comparison of the dropout rates between patients who underwent bariatric surgery within 24 months after treatment (surgical group) and those who received nonsurgical treatment (nonsurgical group)

|  | Surgical group (n=50) | Nonsurgical group (n=271) | P value |
| --- | --- | --- | --- |
| Dropout (%) | 6 (12.0%) | 119 (43.9%) | <0.0001 |

Fisher’s exact test.
