# Supplementary material for: No medication prescription and residential distance from the hospital are important factors associated with nonsurgical weight-loss treatment discontinuance in Japanese patients with high-degree obesity: a retrospective study
Source: BMC Health Serv Res. 2024 Sep 16;24:1078. doi: 10.1186/s12913-024-11474-2 (PMC11407008; doi:10.1186/s12913-024-11474-2)
Supplement: Supplementary file 3 — Supplementary Material 3 [file 12913_2024_11474_MOESM3_ESM.docx]

Supplementary Table 3. The duration between the first visit and second visit and the time with the lowest body weight and BMI after treatment

|  | Non-dropout | Dropout | P value |
| --- | --- | --- | --- |
| Duration between the first and second visit (days) | 35.0 (28.0–44.5) | 35.0 (31.5–49.5) | 0.1592 |
| The time with the lowest body weight and BMI after treatment (months) | 3.0 (1.8–6.0) | 11.0 (5.0–19.0) | <0.0001 |

BMI, body mass index. Wilcoxon rank sum test.
