# Supplementary material for: No medication prescription and residential distance from the hospital are important factors associated with nonsurgical weight-loss treatment discontinuance in Japanese patients with high-degree obesity: a retrospective study
Source: BMC Health Serv Res. 2024 Sep 16;24:1078. doi: 10.1186/s12913-024-11474-2 (PMC11407008; doi:10.1186/s12913-024-11474-2)
Supplement: Supplementary file 4 — Supplementary Material 4 [file 12913_2024_11474_MOESM4_ESM.docx]

Supplementary Table 4. Comparison of the number of comorbidities (type 2 diabetes, hypertension, lipid disorders, hyperuricemia, and thyroid disease) between the non-dropout and dropout groups

|  | Non-dropout | Dropout | P value |
| --- | --- | --- | --- |
| Patients  with lipid disorders  with hyperuricemia  with thyroid disease | 3 (2–3) (n=87)  3 (2–4) (n=44)  4 (2–4) (n=11) | 2 (1–3) (n=52)  3 (2–4) (n=15)  2 (2–2) (n=1) | 0.0264  0.7990  0.2694 |

Wilcoxon rank-sum test.
