# Supplementary material for: No medication prescription and residential distance from the hospital are important factors associated with nonsurgical weight-loss treatment discontinuance in Japanese patients with high-degree obesity: a retrospective study
Source: BMC Health Serv Res. 2024 Sep 16;24:1078. doi: 10.1186/s12913-024-11474-2 (PMC11407008; doi:10.1186/s12913-024-11474-2)
Supplement: Supplementary file 5 — Supplementary Material 5 [file 12913_2024_11474_MOESM5_ESM.docx]

Supplementary Table 5. Comparison of the number of patients with prescriptions from the Diabetes Center in each comorbidity (lipid disorders, hyperuricemia, and thyroid disease) between the non-dropout and dropout groups

|  | Non-dropout | Dropout | P value |
| --- | --- | --- | --- |
| Patients  with lipid disorders  with hyperuricemia  with thyroid disease | 59 (67.8%)  29 (65.9%)  8 (72.7%) | 17 (32.7%)  5 (33.3%)  1 (100%) | 0.0121  0.0367  1.0000 |

Diabetes Center = Center for Diabetes, Endocrine, and Metabolism, Toho University Sakura Medical Center. Fisher’s exact test.
